# Sex differences in dementia with Lewy bodies: an imaging study of neurotransmission pathways

**DOI:** 10.1007/s00259-023-06132-4

**Published:** 2023-02-24

**Authors:** Cecilia Boccalini, Nicolas Nicastro, Debora Elisa Peretti, Silvia Paola Caminiti, Daniela Perani, Valentina Garibotto

**Affiliations:** 1 Vita-Salute San Raffaele University, Milan, Italy; 2 Division of Neuroscience, IRCCS San Raffaele Scientific Institute, Milan, Italy; 3 Laboratory of Neuroimaging and Innovative Molecular Tracers (NIMTlab), Faculty of Medicine, University of Geneva, Geneva, Switzerland; 4 Division of Neurorehabilitation, Department of Clinical Neurosciences, Geneva University Hospitals, Geneva, Switzerland; 5 Division of Nuclear Medicine and Molecular Imaging, Geneva University Hospitals, Geneva, Switzerland; 6 CIBM Center for Biomedical Imaging, Geneva, Switzerland

**Keywords:** Sex, ^123^I-FP-CIT SPECT, Neurotransmission systems, Dementia with Lewy bodies, Molecular connectivity

## Abstract

**Purpose:**

Dementia with Lewy bodies (DLB) is characterized by a wide clinical and biological heterogeneity, with sex differences reported in both clinical and pathologically confirmed DLB cohorts. No research evidence is available on sex differences regarding molecular neurotransmission. This study aimed to assess whether sex can influence neurotransmitter systems in patients with probable DLB (pDLB).

**Methods:**

We included 123 pDLB patients (male/female: 77/46) and 78 control subjects (male/female: 34/44) for comparison, who underwent ^123^I-FP-CIT SPECT imaging. We assessed sex differences in the dopaminergic activity of the nigrostriatal and mesolimbic systems using regional-based and voxel-wise analyses of ^123^I-FP-CIT binding. We tested whether sex-specific binding alterations would also pertain to the serotoninergic and noradrenergic systems by applying spatial correlation analyses. We applied molecular connectivity analyses to assess potential sex differences in the dopaminergic pathways.

**Results:**

We found comparable ^123^I-FP-CIT binding decreases in the striatum for pDLB males and females compared to controls. However, pDLB females showed lower binding in the extrastriatal projections of the nigrostriatal and mesolimbic dopaminergic systems compared to pDLB males. According to the spatial correlation analysis, sex-specific molecular alterations were also associated with serotonergic and noradrenergic systems. Nigrostriatal and mesolimbic systems’ connectivity was impaired in both groups, with males showing local alterations and females presenting long-distance disconnections between subcortical and cortical regions.

**Conclusions:**

Sex-specific differences in ^123^I-FP-CIT binding were found in our cohort, namely, a trend for lower ^123^I-FP-CIT binding in females, significant in the presence of a pDLB diagnosis. pDLB females showed also different patterns of connectivity compared to males, mostly involving extrastriatal regions. The results suggest the presence of a sex-related regional vulnerability to alpha-synuclein pathology, possibly complicated also by the higher prevalence of Alzheimer’s disease co-pathology in females, as previously reported in pDLB populations.

**Supplementary information:**

The online version contains supplementary material available at 10.1007/s00259-023-06132-4.

## Introduction

Nigrostriatal dopaminergic depletion, usually assessed by ^123^I-N-ω-fluoropropyl-2β-carbomethoxy-3β-(4-iodophenyl)nortropane (^123^I-FP-CIT) single-photon emission computed tomography (SPECT), is a hallmark of dementia with Lewy bodies (DLB) [1]. Few studies addressed extrastriatal ^123^I-FP-CIT bindings in DLB patients, reporting lower bindings in the midbrain [2], hypothalamus [3], insula [4], cingulate, and thalamus [5], as compared to controls. Moreover, altered covariance patterns were also reported between thalamic bindings, frontal and parahippocampal projections as well as between cingulate and cortical regions [5]. This suggests long-distance changes in dopaminergic and serotonergic pathways that might be exacerbated by Alzheimer’s disease (AD) co-pathology in DLB [6].

Molecular alterations of ^123^I-FP-CIT binding and connectivity of the neurotransmission pathways are clinically relevant since they are linked to motor, cognitive, and psychiatric features characterizing DLB phenotypes. DLB diagnosis relies on the presence of core clinical features, namely, fluctuating cognition, recurrent visual hallucinations, parkinsonism, and REM sleep behavior disorder (RBD) [1], occurring in the setting of dementia. Not all patients, however, develop all symptoms along the disease course and the sequence of appearance and severity may also vary across patients, leading to a variety of different phenotypes that may be related to the underlying physiopathology. Sex is a basic epidemiological variable that contributes to biological differences in the expression of neurodegenerative diseases [7]. Despite the relevance of sex differences, these are undervalued and understudied in DLB. Only recent reports have shown higher proportions of men with RBD [8] and parkinsonism [8, 9], but not in all cohorts [9], and higher [10, 11] or similar [9] proportions of women with visual hallucinations. Moreover, women have a shorter duration of symptoms before diagnosis, are older, and have lower scores on global cognitive measures, suggesting a more aggressive disease course compared to men, consistent with the more frequent concomitant AD co-pathology [10]. A recent postmortem study showed that sex modifies the associations between AD co-pathology and clinical phenotype in patients with DLB. In the presence of severe AD co-pathology or high tau staging, both women and men showed a reduced likelihood of Lewy body clinical phenotype; however, women had a worse cognitive decline and dementia [12].

In light of the reported clinical and pathological differences, we aim to explore sex differences by using ^123^I-FP-CIT binding in striatal and extrastriatal regions in female and male patients with probable dementia with Lewy bodies (pDLB). Considering the affinity of ^123^I-FP-CIT for transporters involved in different neurotransmission systems (i.e., high-to-low affinity for dopaminergic (DAT), serotonin (SERT), and norepinephrine (NET) transporters, respectively) [13–15], we tested ^123^I-FP-CIT binding in regions innervated by these specific neurotransmitters. Moreover, we investigated the molecular connectivity alterations in nigrostriatal and mesolimbic dopaminergic pathways focusing on sex differences.

## Materials and methods

### Participants

The present study was performed in accordance with the Declaration of Helsinki and its further amendments. Its protocol has been approved by our local Geneva Ethics Committee (NO 2022–01,520) and the subjects’ consent has been waived. We collected all ^123^I-FP-CIT SPECT scans performed at Geneva University Hospitals (Switzerland) between 2013 and 2020 with the same acquisition protocol. Using the most recent clinical diagnostic criteria at the time of diagnosis [1, 16], we retrospectively included 123 subjects (male/female (M/F) = 77/46) with dementia fulfilling the diagnostic criteria of pDLB with available ^123^I-FP-CIT SPECT images. Subjects have been evaluated in the Division of Neurology at Geneva University Hospitals by neurologists with expertise in movement disorders and regular clinical follow-up has been ensured for years in most cases. ^123^I-FP-CIT SPECT was performed as a clinical diagnostic imaging assessment for suspected degenerative parkinsonism or distinction between DLB and AD. Medications known to interfere with ^123^I-FP-CIT binding were suspended at the time of SPECT imaging following current recommendations [17]. For comparison, we included 78 control participants (CTL) (M/F = 34/44) with parkinsonism or tremor not associated with dopaminergic degeneration (e.g., functional tremor/parkinsonism, essential tremor, neuropathic tremor), and with a normal visual and semiquantitative ^123^I-FP-CIT SPECT assessment, already included in previous studies [4, 18, 19]. When several scans were performed for a single subject during the study period, only the first one was considered for the present work.

### ^*123*^*I-FP-CIT SPECT imaging*

^123^I-FP-CIT SPECT was performed at Geneva University Hospitals (Switzerland) with the same acquisition protocol. Patients received 185 MBq of 123I-FP-CIT (ioflupane, DaTSCAN®, GE Healthcare, Glattbrugg, Switzerland) in slow IV injection and Lugol solution or sodium perchlorate to block thyroid uptake. SPECT/CT data acquisition started 4 h after ioflupane injection. The acquisition was performed on a triple-head gamma camera (GCA-9300A/UI Toshiba Medical Systems AG, Oetwil am See, Switzerland) equipped with fan-beam low-energy high-resolution collimators. Details of the acquisition and reconstruction are available in [4, 19]. Pre-processing of SPECT brain images was performed using Statistical Parametric Mapping (SPM12, Wellcome Trust Centre for Neuroimaging, London, UK, https://www.fil.ion.ucl.ac.uk/spm/), running in MATLAB R2018b version 9.5.0 (MathWorks Inc., Sherborn, MA, USA). SPECT images were normalized based on an available ^123^I-FP-CIT template [20]. Intensity-normalized parametric images were generated for each subject using the Image Calculator (ImCalc) function in SPM12. The cerebellar crus II uptake was used as the reference region to calculate the specific binding ratios (SBRs) as [(target region/reference region) − 1]. Asymmetry index (AI) was calculated using ^123^I-FP-CIT putaminal binding ratio (PBR) and the following formula: ((PBR_Higher side_ − PBR_Lower side_)/(PBR_Higher side_ + PBR_Lower side_)) × 2 × 100, to have only positive values expressed in % [21].

The use of the non-specific binding in the cerebellum crus as a reference was based on the observation that it is relatively free from both DAT and SERT [22]. The cerebellum crus has been used in many published works on SERT and thus increases the comparability across results [3, 6, 23, 24]. Two-sample *t*-tests were run comparing females’ and males’ cerebellar uptakes confirming that there are no sex differences in the reference region uptake either in DLB (*p* = 0.537) or HC (*p* = 0.516).

For regions of interest (ROI)-based analysis, we considered ROIs of the nigrostriatal and the mesocorticolimbic dopaminergic pathways, as described elsewhere [25]. All ROIs were derived from the Automated Anatomical Labeling atlas [26]. The putamen and caudate were then subdivided into functional subregions, i.e., dorsal-motor and ventral-limbic divisions [27].

### Statistical analysis

ANOVA tests were used to compare demographics and clinical data between females and males. χ^2^ test was performed for discrete variables, such as clinical symptoms’ frequency. SBR imaging data were compared between females and males using MANCOVA test, including age as a covariate. A post hoc analysis was run, testing for sex × group (DLB vs CTL) interaction effect on regional SBR data. We used SPSS 26.0 software to perform statistical analysis.

### Voxel-wise analysis

To further characterize the results of the regional analysis, a complementary voxel-based analysis was run, assessing the voxel-wise distribution of ^123^I-FP-CIT SBR sex differences. The ^123^I-FP-CIT SBR parametric images were used in two-sample *t*-tests in SPM12, running in MATLAB, to compare pDLB females vs CTL females and pDLB males vs CTL males and, directly pDLB females vs pDLB males. Age was included as a covariate of nuisance. For all voxel-wise analyses, a threshold was set at *p* = 0.005, family-wise error (fwe)-corrected at the cluster level.

We applied the JuSpace toolbox [28] to our dataset to compute Spearman correlation (based on the Neuromorphometrics atlas; adjusted (adj) *p* values, *N* = 10,000 permutations) between *Z*-scores (pDLB females vs CTL females and pDLB males vs CTL males) and the DAT [29], SERT, and NET [30] maps, derived from ^123^I-FP-CIT, ^11^C-DASB, and S,S-^11^C-MRB data, respectively. JuSpace is a comprehensive license-free toolbox running in MATLAB for the integration of positron emission tomography (PET)- and SPECT-derived maps with other brain imaging data (MRI, fMRI), allowing to examine specific neurotransmitter system changes as compared to CTL. Briefly, the PET/SPECT maps were derived from average group maps of different healthy volunteers and consisted of maps of the binding signal intensity across the whole brain. Then, the data to generate a contrast between conditions (patients vs CTL) were entered as input to be correlated with PET-/SPECT-derived maps. The atlas is used to extract mean regional values from the input modalities to be correlated with respective mean values from maps, as a voxel-wise analysis would result in highly inflated degrees of freedom [28].

### Connectivity analysis

Assessment of molecular connectivity between targets of each dopaminergic pathway (nigrostriatal and mesolimbic) was performed via partial correlation analysis computed using MATLAB’s *parcorr* function, as described in previous literature [25, 31–33]. A subject-by-ROI matrix was created for each sex subgroup with pDLB and CTL. The same procedure was applied also on the whole pDLB group vs CTL. The matrices contained the SBRs of the specific ROIs of each network for each subject. Age was included as a nuisance covariate in the partial correlation analysis. The resulting dopaminergic networks were formed by nodes (ROIs), and by edges, represented by the estimated partial correlation coefficient. The procedure for ROIs selection was crucially based only on the cortical targets that showed significant tracer binding in CTL. This resulted in a pool of *N* = 10 ROIs belonging to the nigrostriatal dopaminergic pathway (left/right (L/R) dorsal caudate nucleus, L/R dorsal putamen, L/R inferior frontal gyrus pars opercularis (opIFG), and L/R precentral and postcentral gyri), and *N* = 14 ROIs belonging to the mesolimbic dopaminergic pathway (L/R ventral striatum, L/R hippocampus, L/R amygdala, L/R insula, L/R olfactory cortex (OFC), L/R anterior cingulate cortex (ACC), and L/R middle cingulate cortex (MCC)). Partial correlation coefficients were deemed significant at *p* < 0.01, uncorrected for multiple comparisons.

We applied Fisher’s transformation to the partial correlation coefficients to test whether the strength of each coefficient (between nodes-dopaminergic networks) differed between groups. A *Z*-test was used to test for significant changes in partial correlation coefficients. All the results were set at a statistical threshold of *p* < 0.01, uncorrected for multiple comparisons. Since the sample sizes of the target population and the reference group of CTL should be similar to ensure a robust statistical comparison of connectivity metrics, sex-matched DLB subgroups were randomly selected for comparison, with the same number of the same-sex CTL group (F/M = 44/34).

In male and female patients, we calculated the percentage of altered molecular connections in each network to quantify the severity of molecular connectivity alterations. Thus, we computed the number of altered connections divided by the total number of connections of each network to obtain the percentage of altered molecular connectivity. Then, we compared the number of altered molecular connections between male and female patients for each network through the χ^2^ test.

## Results

Clinical and demographic features of the whole pDLB group (M/F = 77/46) are reported in Table 1. Female and male patients did not differ regarding clinical and demographic features, except for age with women being older than men at the time of diagnosis.

**Table 1:** Demographic and clinical features of the DLB sample

	Whole DLB sample	DLB males	DLB females	Statistic *p* value
Number	123	77	46	
Age, year	77.15 ± 6.50	76.14 ± 6.46	78.84 ± 6.27	**0.025**^**a**^
Disease duration, year	1.81 ± 1.72	1.92 ± 1.68	1.62 ± 1.80	0.397^a^
DaTSCAN, N positive cases	112 (91%)	70 (91%)	42 (91%)	0.940^b^
Putamen asymmetry index (%)	22.02 ± 14.76	20.87 ± 13.55	23.94 ± 16.57	0.265^a^
Clinical symptoms				
Parkinsonism, frequency	90.2%	88.6%	93.1%	0.519^b^
Visual hallucinations, frequency	73.2%	71.6%	75.8%	0.684^b^
Cognitive fluctuations, frequency	56.1%	62.2%	44.8%	0.128^b^
REM-sleep behavior disorder, frequency	17.2%	21.3%	9.4%	0.147^b^

Abbreviations: *DLB*, dementia with Lewy bodies; *N*, number

^a^Two-sample *t*-test

^b^Chi-square test

Significant *p*-values are reported in boldface

### *Sex differences in *^*123*^*I-FP-CIT SPECT imaging*

**Fig. 1:**
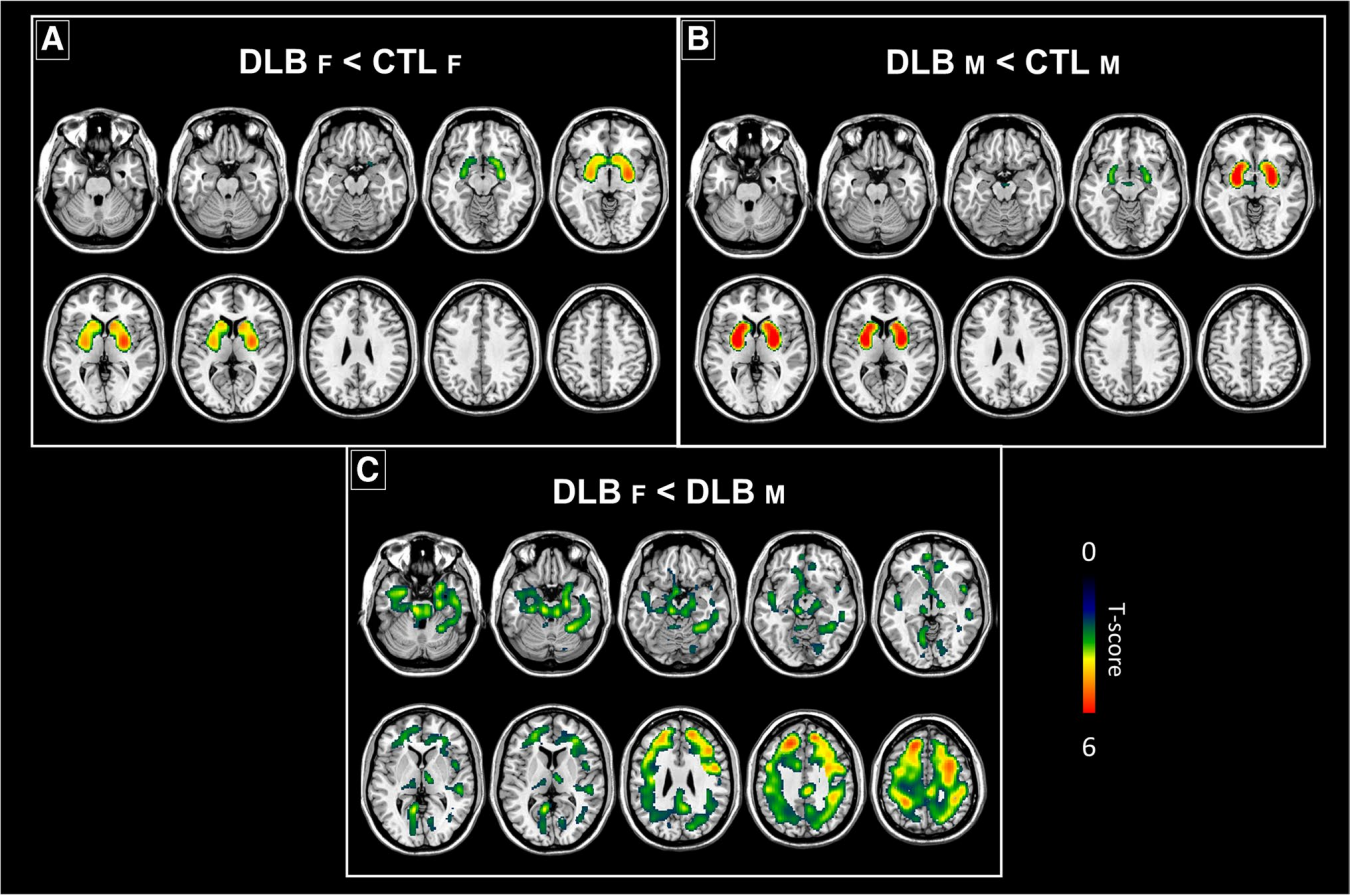
Voxel-wise sex differences in ^123^I-FP-CIT binding. Panels **A** and **B** show the distribution of voxel-wise differences in ^123^I-FP-CIT SBR for each sex group, resulting from statistical comparison with sex-matched CTL. Panel **C** shows the results from the direct voxel-wise comparison between females and males with DLB, after adjusting for age. The magnitude of the difference is reported by means of *t*-score. Abbreviations: DLB, dementia with Lewy bodies; CTL, controls; F, females; M, males

The pDLB male and female cohorts showed significant reductions of ^123^I-FP-CIT-SPECT bindings in comparison with CTL (Fig. 1 and Table 2). Voxel-wise analysis assessing differences between each sex group vs sex-matched CTL showed severe alterations of DAT binding density in the basal ganglia for both pDLB females and males (Fig. 1). The binding reductions (*t*-scores) were higher for pDLB males than females as visible in Fig. 1B.

**Table 2:** Regional analysis of ^123^I-FP-CIT-SPECT imaging bindings in the male and female DLB patients

	DLB males *N* = 77	CTL males *N* = 34	Statistic DLB_M_ vs CTL_M_	DLB females *N* = 46	CTL females *N* = 44	Statistic DLB_F_ vs CTL_F_	Statistic CTL_M_ vs CTL_F_	Statistic DLB_M_ vs DLB_F_
Nigrostriatal system ROIs								
Left dorsal caudate	3.68 ± 1.97	6.11 ± 2.38	***p* < *0.001***	3.46 ± 1.79	6.05 ± 2.38	***p* < *0.001***	*p* = *0.826*	*p* = *0.597*
Right dorsal caudate	3.39 ± 1.80	5.63 ± 2.29	***p* < *0.001***	3.00 ± 1.59	5.65 ± 2.28	***p* < *0.001***	*p* = *0.864*	*p* = *0.275*
Left dorsal putamen	4.93 ± 2.15	8.05 ± 2.09	***p* < *0.001***	4.72 ± 2.30	7.28 ± 2.07	***p* < *0.001***	*p* = *0.063*	*p* = *0.499*
Right dorsal putamen	5.00 ± 2.29	8.07 ± 2.11	***p* < *0.001***	4.61 ± 2.22	7.32 ± 2.20	***p* < *0.001***	*p* = *0.079*	*p* = *0.241*
Left opIFG	0.85 ± 0.59	0.93 ± 0.56	*p* = *0.785*	0.62 ± 0.36	0.71 ± 0.38	*p* = *0.814*	***p* = *0.016***	***p* = *0.028***
Right opIFG	0.69 ± 0.52	0.67 ± 0.47	*p* = *0.817*	0.54 ± 0.36	0.63 ± 0.54	*p* = *0.546*	*p* = *0.482*	*p* = *0.088*
Left precentral gyrus	1.01 ± 0.60	0.96 ± 0.48	*p* = *0.808*	0.67 ± 0.40	0.83 ± 0.39	*p* = *0.156*	***p* = *0.001***	***p* < *0.001***
Right precentral gyrus	0.89 ± 0.56	0.86 ± 0.49	*p* = *0.678*	0.64 ± 0.42	0.76 ± 0.40	*p* = *0.253*	*p* = *0.060*	***p* = *0.006***
Left postcentral gyrus	0.94 ± 0.54	0.88 ± 0.46	*p* = *0.613*	0.69 ± 0.37	0.79 ± 0.37	*p* = *0.296*	***p* = *0.018***	***p* = *0.004***
Right postcentral gyrus	0.85 ± 0.54	0.77 ± 0.47	*p* = *0.416*	0.63 ± 0.37	0.79 ± 0.41	*p* = *0.093*	*p* = *0.907*	***p* = *0.008***
Mesolimbic system ROIs								
Left ventral striatum	4.14 ± 2.01	6.14 ± 2.18	***p* = *0.001***	3.57 ± 1.61	5.69 ± 2.12	***p* < *0.001***	*p* = *0.241*	*p* = *0.132*
Right ventral striatum	3.93 ± 1.93	5.90 ± 2.15	***p* < *0.001***	3.37 ± 1.51	5.51 ± 2.03	***p* < *0.001***	*p* = *0.360*	*p* = *0.109*
Left amygdala	1.48 ± 0.79	1.57 ± 0.58	*p* = *0.533*	1.13 ± 0.51	1.43 ± 0.64	*p* = *0.101*	*p* = *0.256*	***p* = *0.010***
Right amygdala	1.67 ± 0.84	2.01 ± 0.80	*p* = *0.117*	1.34 ± 0.56	1.68 ± 0.66	***p* = *0.046***	*p* = *0.055*	***p* = *0.018***
Left hippocampus	1.02 ± 0.59	0.98 ± 0.51	*p* = *0.412*	0.78 ± 0.37	0.88 ± 0.78	*p* = *0.904*	*p* = *0.256*	***p* = *0.025***
Right hippocampus	1.18 ± 0.68	1.20 ± 0.56	*p* = *0.558*	0.93 ± 0.43	1.09 ± 0.50	*p* = *0.467*	*p* = *0.266*	*p* = *0.057*
Left parahippocampus	0.56 ± 0.51	0.50 ± 0.41	*p* = *0.506*	0.31 ± 0.30	0.39 ± 0.38	*p* = *0.957*	*p* = *0.088*	***p* = *0.006***
Right parahippocampus	0.83 ± 0.56	0.76 ± 0.49	*p* = *0.276*	0.51 ± 0.33	0.60 ± 0.39	*p* = *0.881*	***p* = *0.024***	***p* = *0.001***
Left insula	1.22 ± 0.67	1.36 ± 0.61	*p* = *0.454*	0.99 ± 0.38	1.24 ± 0.56	*p* = *0.225*	*p* = *0.346*	***p* = *0.043***
Right insula	1.01 ± 0.58	1.05 ± 0.55	*p* = *0.872*	0.74 ± 0.34	0.95 ± 0.52	*p* = *0.376*	*p* = *0.273*	***p* = *0.007***
Left olfactory cortex	1.29 ± 0.76	1.41 ± 0.76	*p* = *0.946*	0.91 ± 0.50	1.34 ± 0.75	*p* = *0.095*	*p* = *0.491*	***p* = *0.006***
Right olfactory cortex	1.41 ± 0.85	1.72 ± 0.88	*p* = *0.413*	1.07 ± 0.50	1.60 ± 0.76	***p* = *0.020***	*p* = *0.402*	***p* = *0.028***
Left anterior cingulate	0.90 ± 0.62	0.90 ± 0.55	*p* = *0.707*	0.65 ± 0.37	0.68 ± 0.43	*p* = *0.497*	***p* = *0.018***	***p* = *0.021***
Right anterior cingulate	1.10 ± 0.68	1.10 ± 0.64	*p* = *0.946*	0.81 ± 0.42	0.79 ± 0.43	*p* = *0.405*	***p* = *0.007***	***p* = *0.009***
Left middle cingulate	1.16 ± 0.73	1.23 ± 0.66	*p* = *0.865*	0.82 ± 0.43	1.00 ± 0.61	*p* = *0.435*	***p* = *0.019***	***p* = *0.006***
Right middle cingulate	1.18 ± 0.71	1.28 ± 0.67	*p* = *0.783*	0.84 ± 0.43	1.09 ± 0.66	*p* = *0.219*	*p* = *0.053*	***p* = *0.005***
Other ROIs								
Left thalamus	1.03 ± 0.64	1.10 ± 0.63	*p* = *0.975*	0.72 ± 0.36	1.04 ± 0.59	*p* = *0.107*	*p* = *0.453*	***p* = *0.011***
Right thalamus	1.20 ± 0.67	1.26 ± 0.63	*p* = *0.898*	0.92 ± 0.37	1.22 ± 0.61	*p* = *0.113*	*p* = *0.700*	***p* = *0.020***
Left globus pallidus	4.64 ± 1.95	7.01 ± 2.11	***p* < *0.001***	4.29 ± 1.84	6.42 ± 2.06	***p* < *0.001***	*p* = *0.142*	*p* = *0.287*
Right globus pallidus	5.15 ± 2.31	8.38 ± 2.41	***p* < *0.001***	4.79 ± 2.22	7.65 ± 2.36	***p* < *0.001***	*p* = *0.121*	*p* = *0.311*

Abbreviations: *DLB*, dementia with Lewy bodies; *CTL*, controls; *N*, number; *opIFG*, inferior frontal gyrus pars opercularis; *ROIs*, regions of interest

All *p*-values are reported in italic. Significant *p*-values are reported in italic and boldface

When we directly compared voxel-wise ^123^I-FP-CIT binding of pDLB females vs pDLB males, we found reduced bindings in the thalamus, amygdala, insula, hippocampus, parahippocampus, ACC, MCC, orbitofrontal cortex, middle frontal gyrus, precentral and postcentral gyri, as well as the inferior parietal lobule in pDLB females (Fig. 1C). No differences were found with the opposite contrast.

The ROI-based analysis revealed more significant SBR decreases for pDLB females than males in several ROIs depending on the nigrostriatal (left opIFG, bilateral precentral and postcentral gyri (*p* < 0.05)), mesolimbic dopaminergic, and serotonergic systems, namely, the bilateral amygdala, insula, hippocampus, parahippocampus, OFC, ACC, and MCC (*p* < 0.05). Females showed more significant SBR decreases also in the bilateral thalamus as compared to males. In most of the aforementioned regions showing significantly lower SBR in females pDLB, we also found a trend toward a lower SBR in females CTL (Table 2). In CTL, significant sex differences were found only in the left opIFG, left precentral gyrus, left postcentral gyrus, ACC, and MCC. Results are fully reported in Table 2. Although both sex and group (pDLB vs CTL) have main effects on SBR, no significant interaction effect between group and sex was found, indicating that sex effect on regional SBR does not differ between pDLB and CTL, after controlling for age. This confirms that sex and group are both significant and independent predictors of SBR decreases in the investigated ROIs.

**Fig. 2:**
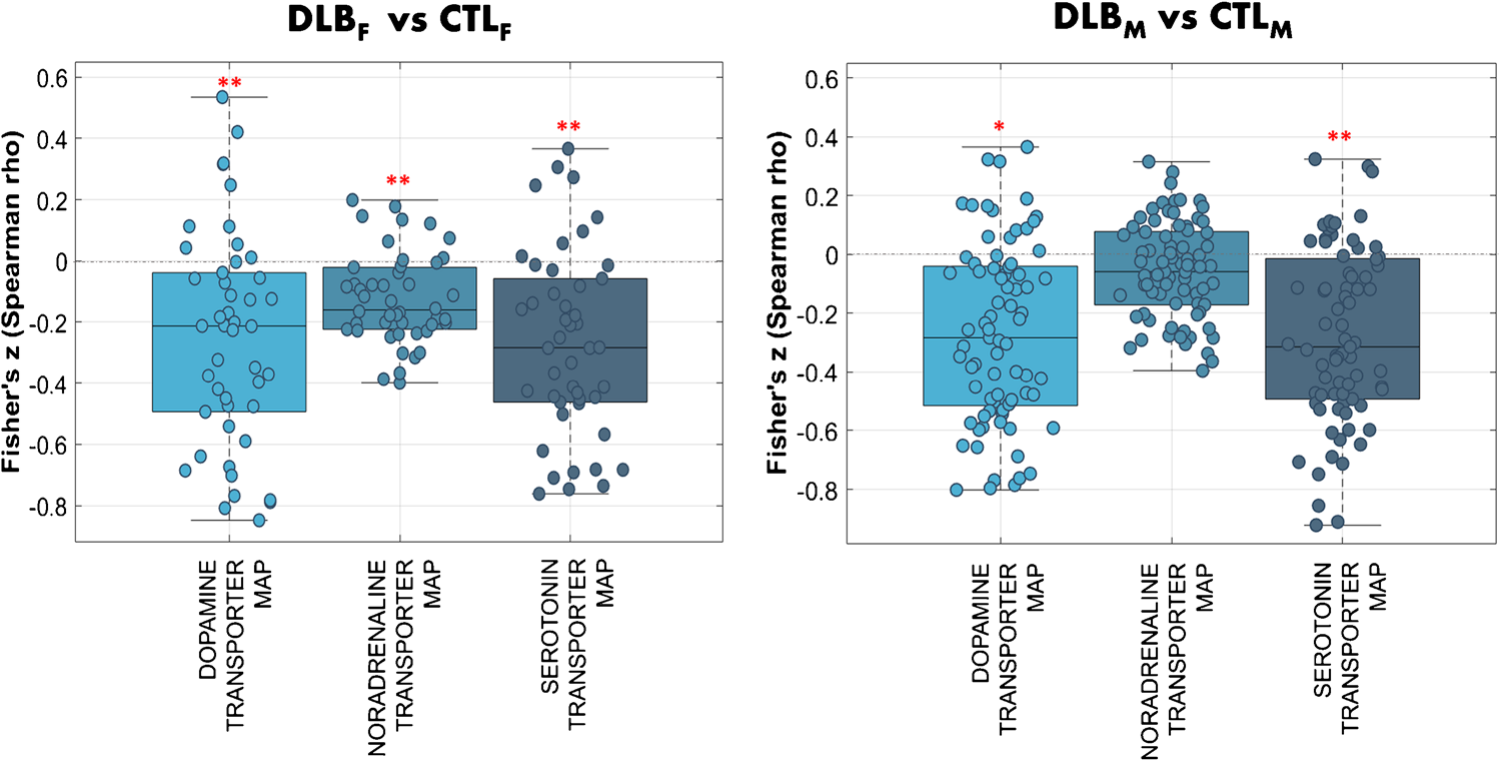
Results of spatial correlation analysis with neurotransmitter maps for DLB females and males. Fisher’s *z*-transformed correlation coefficients with respective neurotransmitter maps are displayed for each subject and contrast. ^123^I-FP-CIT binding alterations in DLB females and males as compared to controls are significantly associated with the topographies of DAT and SERT. ^123^I-FP-CIT binding alterations in DLB females are associated with NET. Error bars represent the parametric 95% confidence interval of the mean. Exact permutation-based *p* values (10,000 permutations) were computed for all analyses: ** indicates *p* values < 0.001; whereas * indicates *p* values < 0.005. See text for details. Abbreviations: DLB, dementia with Lewy bodies; F, females; M, males; CTL, controls

Since ^123^I-FP-CIT can be used for the evaluation of other monoaminergic systems beyond DAT [13–15], especially in extrastriatal regions, we assessed the mapping of ^123^I-FP-CIT binding alterations with different neurotransmission templates, using JuSpace toolbox [28–30]. We showed a significant spatial correlation with the DAT alterations (*p* < 0.005) for both pDLB groups compared to CTL (*z*-scores). In addition, neurotransmitter alterations significantly correlated with SERT receptor map in both pDLB females and males (*p* < 0.001) and with NET receptor map only in pDLB females (*p* < 0.001). Fig. 2 shows Fisher’s *z*-transformed correlation coefficients with respective neurotransmitter maps for each subject (individual points) and contrast.

### Sex differences in molecular connectivity

#### Nigrostriatal dopaminergic system

Considering the whole pDLB group, several connectivity alterations (22.5%) affected the nigrostriatal system as compared to CTL. These alterations involved subcortical connections between the putamen and caudate nucleus, with a loss of connectivity with the contralateral striatum and increased pathological connections with the ipsilateral structures as compared to CTL. Moreover, pDLB lost subcortical-cortical connections between the striatum and the postcentral and precentral gyri as compared to CTL.

pDLB males’ connectivity pattern showed only short-distance local subcortical alterations within the striatum. The pDLB females showed long-distance alterations between the caudate nucleus and precentral and postcentral gyri, and between opIFG and precentral gyrus. However, a direct comparison between males’ and females’ altered connections did not show any significant difference, i.e., females (20%) and males (10%) (χ^2^ = 1.56, *p* = 0.21).

#### Mesolimbic dopaminergic system

pDLB patients showed 6% of altered connections as compared to CTL. Specifically, there was altered connectivity between the amygdala and the ipsilateral ventral striatum, the MCC, and ACC. In turn, ACC showed altered connectivity with the hippocampus.

pDLB females and males did not differ in the number of connectivity alterations (χ^2^ = 0.14, *p* = 0.69). However, we found sex differences in the connectivity patterns: altered connectivity patterns of pDLB females involved limbic structures, i.e., the amygdala, insula, OFC, MCC, and ACC, whereas connectivity alterations in pDLB males were more extensive, also affecting the hippocampus.

See Supplemental Fig. 1 for the matrices resulting from the connectivity analyses.

## Discussion

The large variability of phenotypes in DLB patients [34] suggests that multiple modulating factors, including sex, might influence clinical manifestations. This study provided the first evaluation of the influence of sex on molecular endophenotypes in pDLB, considering neurotransmission impairments and molecular connectivity.

We found comparable ^123^I-FP-CIT binding decreases in dopaminergic systems in pDLB males and females in comparison to CTL. However, females showed more reduced extrastriatal binding than males (Fig. 1C). As a trend toward similar sex differences was also found in the CTL group when comparing males and females, lower binding levels in these regions might represent a sex-related vulnerability to pathology. Connectivity of the nigrostriatal and mesolimbic systems was affected in both sex groups, but with different patterns. In line with the more extensive neurodegeneration hypothesis, beyond the striatal regions, pDLB females showed more long-distance connectivity alterations between subcortical and cortical regions belonging to the dopaminergic systems, in comparison to pDLB males. This study also revealed different molecular dysfunctions linked to monoaminergic transporters’ availability.

From a clinical standpoint, our data are in line with a male predominance in pDLB, as 62% of patients in our cohort were men. The male predominance is lower compared to previous works [7, 10]; however, it is consistent with the age of our cohort. Sex differences in prevalence are expected to be lower with increasing age (75–80) [10, 35], maybe in association with the drop in neuroprotective effects of estradiol [36]. In addition, the more frequent concomitant AD pathology in females should be considered [9, 10]. AD co-pathology leads to more pronounced typical AD symptoms [37], which can introduce a risk of underdiagnosing women with DLB in favor of AD [9, 10]. Moreover, pDLB females in our cohort are older than males at the time of diagnosis [9] in line with the evidence that women meet clinical criteria for pDLB at an older age and after a longer latency from cognitive onset [38]. Despite women being less likely to meet clinical diagnostic criteria for pDLB, when symptoms are present, the clinical severity is not milder than in men [9]. Accordingly, we did not find any significant differences in terms of clinical core features (frequency of parkinsonism, RBD, cognitive fluctuations, and visual hallucinations were similar in our cohorts) (Table 1). Here we show relevant data on the sex-specific alterations in ^123^I-FP-CIT binding linked to other monoaminergic systems with sex differences to be considered for future studies assessing clinical phenotypes.

From the molecular perspective, both sex groups were characterized by loss of striatal DAT binding, a common hallmark of DLB [39], and also extrastriatal binding, in line with previous studies [2, 40]. Moreover, we highlighted a tendency for sex-specific differences in ^123^I-FP-CIT binding, also in CTL, that become significant in presence of pDLB diagnosis (Fig. 1A and B). The comparison with CTL revealed a greater decrease in striatal bindings for pDLB males. Conversely, pDLB females showed lower ^123^I-FP-CIT binding in cortical targets of the nigrostriatal dopaminergic system, i.e., middle frontal gyrus, precentral and postcentral gyri, but also in the extrastriatal regions belonging to the mesolimbic dopaminergic systems, i.e., amygdala, hippocampus, parahippocampus, insula, and cingulate gyrus compared to pDLB males (Fig. 1C). Notably, the trend toward sex differences in the same extrastriatal regions in CTL suggests that sex vulnerabilities may not reflect a sole effect of DLB pathology, but a multifactorial mechanism which is present, albeit to a lesser extent, even in the absence of a neurodegenerative process. In pDLB, the females’ lower binding in the cortical regions involved in the nigrostriatal dopaminergic system is in line with the greater incidence of parkinsonism reported in our female sample as compared to males (Table 1). The great impairment of the nigrostriatal system was also supported by the sex difference in connectivity patterns involving connections between the frontoparietal cortical motor and striatal regions, which was only observed for females.

Sex molecular differences emerged also in the mesolimbic structures’ binding, with a sex-specific vulnerability disfavoring females. A lower ^123^I-FP-CIT extrastriatal binding in females than males is found here also in CTL, without reaching the threshold for significant differences, as happened instead for pDLB. The limbic system is vulnerable to alpha-synuclein pathology and potentially explains autonomic and affective dysfunctions in DLB patients [41]. Notably, a recent study found a similar limbic dopaminergic vulnerability in females with PD supporting the higher incidence of severe psychopathological states [25]. Taken together, these results suggest a common sex-dependent vulnerability of the mesolimbic system in alpha-synucleinopathies [25]. In the absence of an interaction effect on the molecular binding in some regions applying the ROI-based approach, one can speculate that a sex-specific vulnerable ground could be present even in a healthy population [42]. Previous studies focused on sex differences in striatal DAT reporting healthy women to have higher striatal binding compared with healthy men that tend to disappear with aging [43]. Albeit the sex-related differences are controversial [19] and mostly based on conventional regions of interest-based analysis [44], our results extend the literature supporting that sex differences in ^123^I-FP-CIT  extrastriatal binding may exist in healthy populations and can influence the disease-related reduction in DAT. Limbic structures are highly interconnected and sensitive to stress and sex hormones, with high-stress vulnerability in females even in the healthy population [45]. Then, the neurodegenerative processes might differently impact males and females depending also on several factors, including genetics, environmental exposure, and reproductive hormones.

Noteworthy, alpha-synuclein pathology plays a key role in the neurotransmitter impairment affecting different clinically relevant systems, such as the dopaminergic, serotonergic, and noradrenergic ones [46], for which ^123^I-FP-CIT binding has high-to-low affinity, respectively [13–15]. To explore the sex-specific alterations in ^123^I-FP-CIT binding linked to other neurotransmitter systems beyond DAT, we applied spatial association analysis with specific neurotransmitter maps [28]. We found ^123^I-FP-CIT binding alterations associated also with SERT in both sex groups and NET only in females (Fig. 2), which is consistent with the affinity of the tracer for transporters involved in these different neurotransmission systems [13–15] and supports their clinical relevance. Monoaminergic neurotransmitter alterations in DLB have been related to affective symptoms, such as depression and psychosis [47, 48]. Postmortem studies identified the decreased serotonergic neurotransmission as the main monoaminergic etiology of depression in DLB, whereas impaired dopaminergic activity across the mesolimbic system might clinically account for psychosis [48]. Noradrenergic deficiency is also of great magnitude, occurs early, and shows a strong correlation with disease severity in DLB, involving autonomic dysfunction, sleep disturbances, and cognitive decline [49]. Moreover, there is evidence that the presence of AD plaques and tangles in addition to Lewy body pathology further impairs noradrenergic neurotransmission in the locus coeruleus in DLB [48]. Of relevance, we found that sex-related ^123^I-FP-CIT binding alterations were associated with the NET system only in pDLB females, who usually present a great burden of AD pathology [10, 12]. A recent study explored the influence of concomitant AD pathology in DLB on both striatal and extrastriatal ^123^I-FP-CIT binding, highlighting lower extrastriatal binding in the group with co-pathology [6]. The authors supported more extensive neurodegeneration, including the dopaminergic and serotonergic systems, in mixed pathology, possibly due to a synergistic effect of concomitant AD pathology.

Limitations of the present study include its retrospective design which cannot control for selection biases and the absence of an in-depth clinical, neuropsychological, psychiatric evaluation, and MRI imaging that would have allowed the assessment of sex effects on the clinical-molecular correlations and the partial volume effect correction, respectively. Moreover, information on RBD was based on participant reports, which can be less reliable than more objective measures, such as polysomnography. Our analyses did not include healthy aging individuals as reference, but a control population of subjects with non-degenerative conditions and normal SPECT, validated to serve as control reference in previous studies [4, 18, 19]. We observed sex-specific correlations using the JuSpace toolbox also for the serotonergic and noradrenergic systems, which should be taken with caution considering that the mean correlations were relatively modest and that we cannot exclude that an association between low differences and low binding partially contributes to the correlation values we found. Lastly, given the high risk for misdiagnosis and underdiagnosis of DLB, especially in females, it would have been important to have pathological confirmation or amyloid and tau biomarkers to confirm the co-pathology.

## Conclusion

The present results suggest sex-specific patterns of neurotransmission vulnerability in pDLB. A more extensive depletion of extrastriatal dopaminergic binding, but also serotonergic and noradrenergic dysfunction, in pDLB females than males, is in line with the high prevalence of AD pathology and great clinical disease burden in females, as previously reported [10, 12]. Given the previous evidence of co-pathology as a possible driver of sex differences [10, 12], further sex studies combining in vivo molecular imaging with amyloid and tau data are warranted to confirm our findings. Moreover, our results advocate future sex-related studies on the serotonergic and noradrenergic systems in DLB, using specific radiotracers, in large patient samples. Insights into the disease-specific sex effect on monoaminergic deficits might have implications for disease management in a precision medicine approach.

## Supplementary Information

ESM 1 Supplemental figure 1 Dopaminergic connectivity results in the whole group, and separately in females and males. The matrices represent the significant differences obtained when comparing partial correlation coefficients between DLB < CTL, DLB males < CTL males and DLB females < CTL females, in the dopaminergic networks. The color bar displays the Z scores’ values to compare partial correlation coefficients’ strengths. Altered connections are presented: in red, the increased and in blue, the decreased connections compared with CTL. Abbreviations: DLB, dementia with Lewy bodies; CTL, controls; L, left; R, right; DC, dorsal caudate nucleus; DP, dorsal putamen, VS, ventral striatum, HIP, hippocampus; AMY, amygdala; ACC, anterior cingulate cortex, MCC, middle cingulate cortex; OFC, olfactory cortex; IFGop, inferior frontal gyrus pars opercularis; PreCG, precentral gyrus; PoCG, postcentral gyrus (PNG 270 kb)

High resolution image (TIF 4122 kb)

## Data Availability

The datasets generated during and/or analyzed during the current study are available from the corresponding author upon reasonable request.
