# Supplementary figures and images for: Sex differences in dementia with Lewy bodies: an imaging study of neurotransmission pathways
Source: Eur J Nucl Med Mol Imaging. 2023 Feb 24;50(7):2036–46. doi: 10.1007/s00259-023-06132-4 (PMC10199852; doi:10.1007/s00259-023-06132-4)

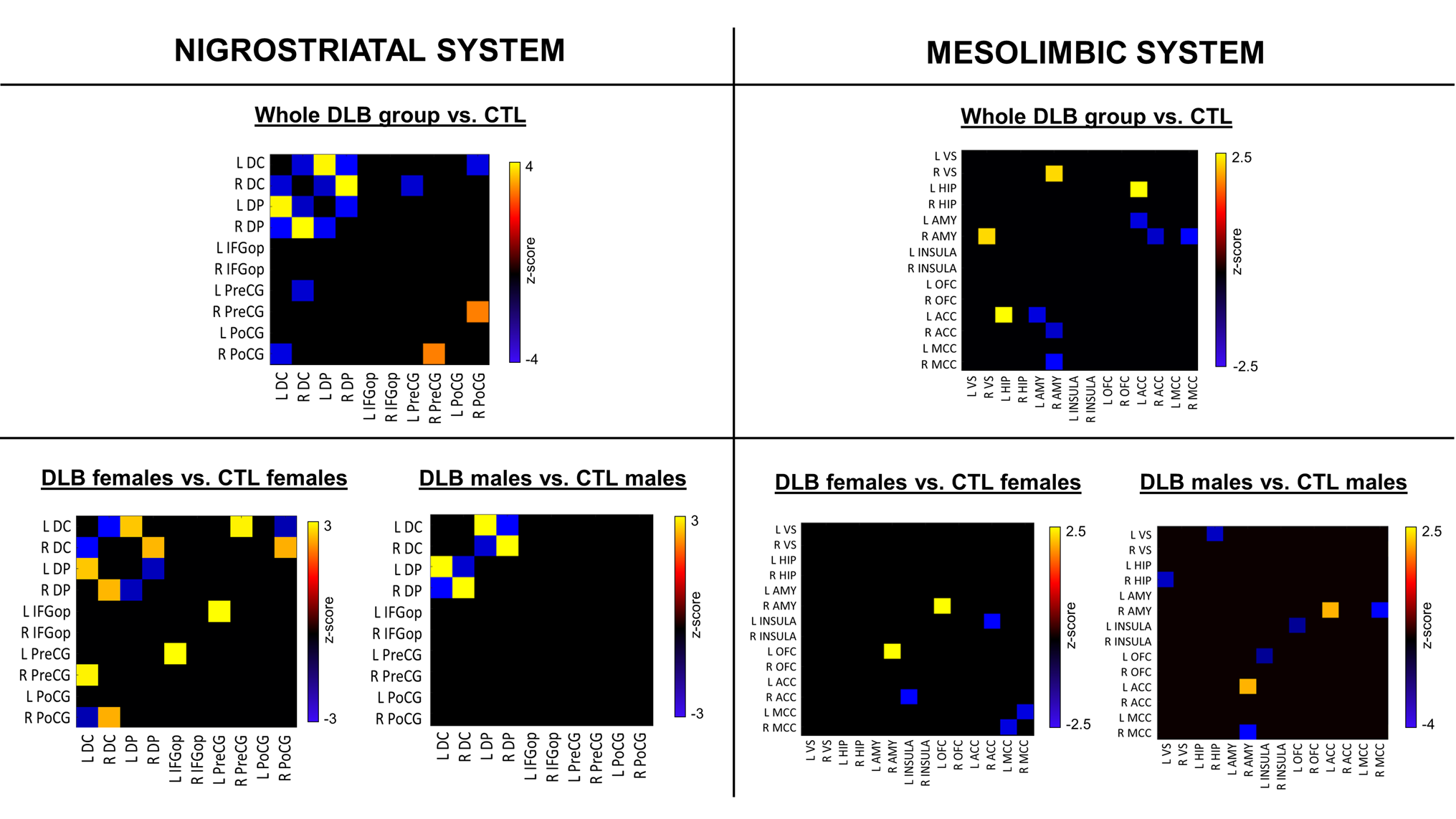

Supplement: Supplementary file 1 — Supplemental figure 1 Dopaminergic connectivity results in the whole group, and separately in females and males. The matrices represent the significant differences obtained when comparing partial correlation coefficients between DLB < CTL, DLB males < CTL males and DLB females < CTL females, in the dopaminergic networks. The color bar displays the Z scores’ values to compare partial correlation coefficients’ strengths. Altered connections are presented: in red, the increased and in blue, the decreased connections compared with CTL. Abbreviations: DLB, dementia with Lewy bodies; CTL, controls; L, left; R, right; DC, dorsal caudate nucleus; DP, dorsal putamen, VS, ventral striatum, HIP, hippocampus; AMY, amygdala; ACC, anterior cingulate cortex, MCC, middle cingulate cortex; OFC, olfactory cortex; IFGop, inferior frontal gyrus pars opercularis; PreCG, precentral gyrus; PoCG, postcentral gyrus (PNG 270 kb) [file 259_2023_6132_Fig3_ESM.png]

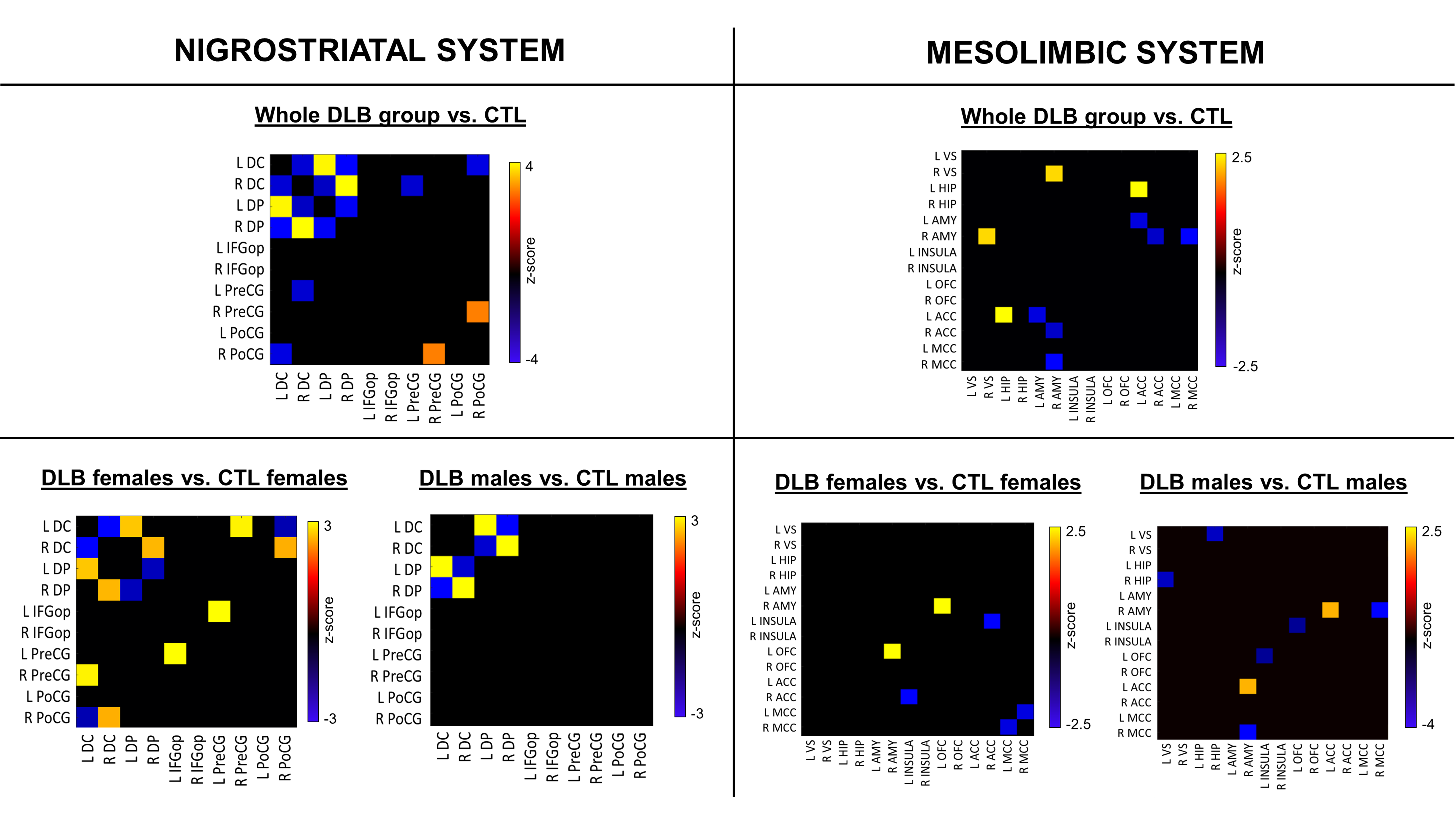

Supplement: Supplementary file 2 — High resolution image (TIF 4122 kb) [file 259_2023_6132_MOESM1_ESM.tif]
